# Fetal blood flow measured using phase contrast MRI-comparison of image quality and flow volume at 1.5T with 3.0T

**DOI:** 10.1186/1532-429X-17-S1-O60

**Published:** 2015-02-03

**Authors:** Beverly Tsai-Goodman, Mike Seed, Christopher Macgowan

**Affiliations:** 1 University of Toronto, Toronto, ON, Canada; 2 The Hospital for Sick Children, Toronto, ON, Canada; 3 Royal Hospital for Sick Children, Bristol, UK

## Background

Ultrasound is commonly used for the antenatal assessment of a fetus and it can provide functional and haemodynamic data in addition to anatomical details. The accuracy of the former is dependant on angle of insonation, maternal habitus and fetal position. Phase contrast cardiovascular magnetic resonance (PC CMR) has emerged as a clinical tool for blood flow quantification but its use in the foetus has been hampered by the need for gating with the fetal heart beat. Previously described metric optimized gating (MOG) technique has been successfully used to measure fetal blood flow in late gestation foetuses on a 1.5T MRI magnet. However, there is increasing interest in performing foetal cardiac imaging using 3.0T MRI. We describe our pilot data of fetal blood flow measured in 3.0T MRI using MOG technique.

## Methods

Fetal blood flows were quantified in 5 subjects at late gestational age (35-38 weeks) of which were two normal and three pregnancies with ventricular size discrepancy. The data were obtained on 1.5T and 3.0T (Siemens Avanto and TRIO, respectively) within the same day using a previously described protocol. After MOG reconstruction of PC MR data, blood flow was quantified by using Q flow (Medis, NL) and adjusted for fetal mass. Reproducibility of flow measurements at the two field strengths was assessed by Pearson correlation coefficient (R^2^), linear regression and Bland Altman analysis.

## Results

**Figure 1:**
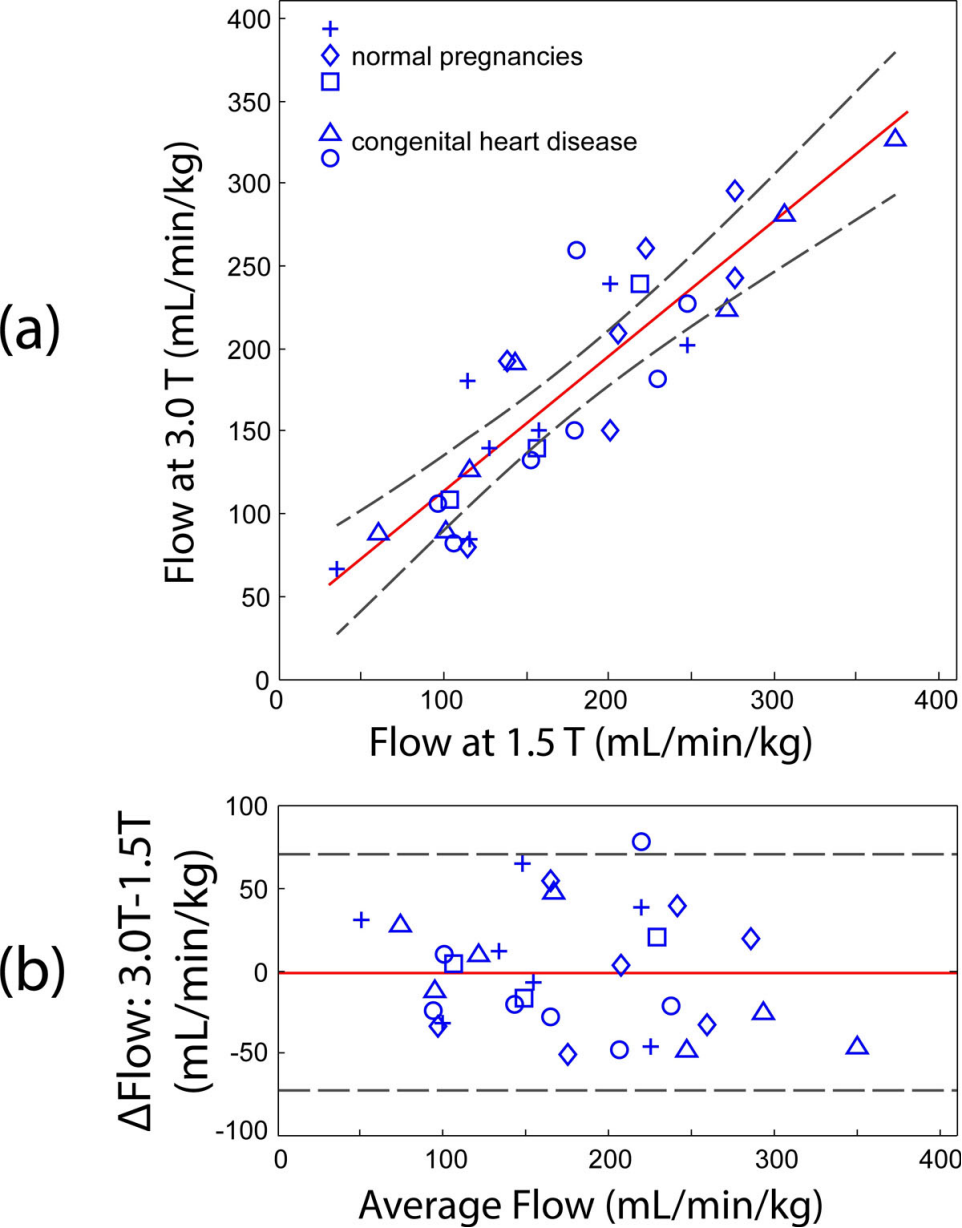
(a) Comparison of fetal flows (5 subjects) measured at 3.0T versus 1.5T using PC CMR with MOG. Solid red line = linear regression; Dashed lines = 95% confidence interval (CI) (b) Bland-Altman analysis of data from (a). Solid red line = mean; Dashed = 95% CI

**Figure 2:**
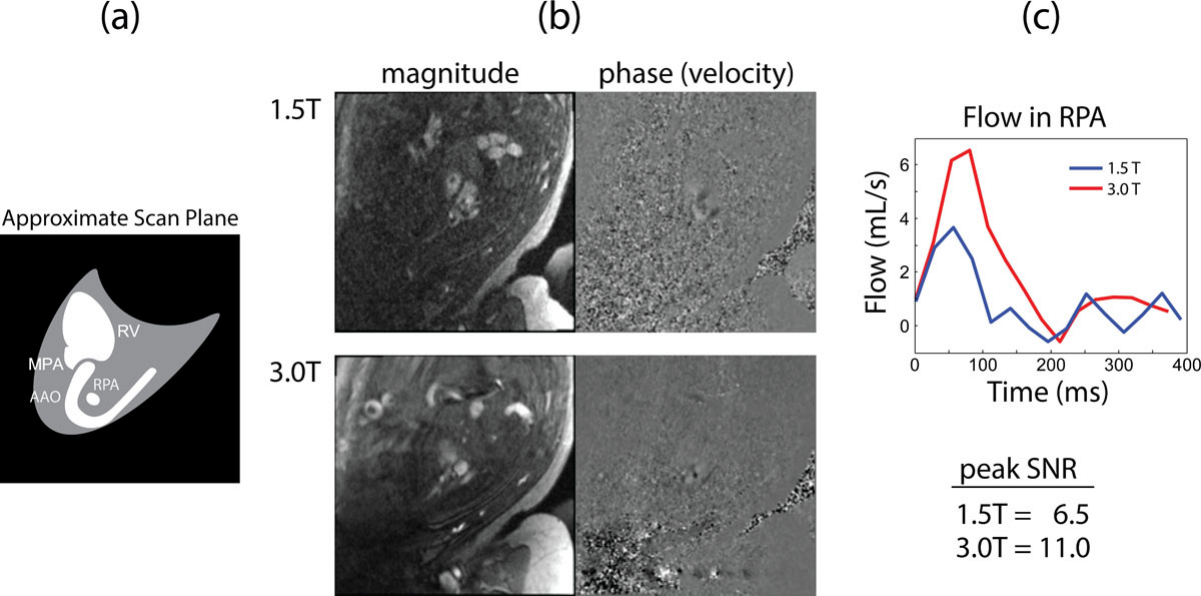
Comparison of PC CMR of the RPA at 1.5 T and 3.0 T. (a) Diagram of slice orientation through the fetal anatomy showing major vascular landmarks. (b) Magnitude and phase (velocity) data at 1.5 and 3.0 T, demonstrating superior SNR and anatomical visualization at 3.0 T. (c) Corresponding flow waveforms obtained from the RPA at 1.5 T (blue) and 3.0 T (red), and peak SNR (per pixel) for the RPA. RV = Right ventricle.

PC CMR flow measurements were obtained in 31 of 35 target vessels. A correlation plot showed strong agreement between corresponding measurements at each field strength (R^2^ = 0.78, slope = 0.83 ± 0.11) with a mean and 95% confidence interval (C.I.) of -1 ml/min/kg and 71 ml/min/kg, respectively (Fig. 1) . Across all measurements, SNR at 3.0 T was increased by 165 ± 16 % relative to 1.5 T and this was more noticeable the smaller the vessel (Fig. 2).

## Conclusions

This is the first pilot study to measure fetal blood flow using MOG in 3.0T field strength. The flow data obtained were in good correlation with those measured at 1.5T. Due to increase SNR and therefore better visualization of the smaller vessels, the MOG algorithm and the final flow measurements were much improved.

## Funding

None.

